# Large parathyroid adenomas: Potential mechanisms to reconcile adenoma size and disease phenotype

**DOI:** 10.3389/fendo.2023.1009516

**Published:** 2023-02-02

**Authors:** Arti Bhan, Shobana Athimulam, Poonam Kumari, Rimesh Pal, Sanjay Kumar Bhadada, Bernard C. Cook, Shijing Qiu, Sudhaker D. Rao

**Affiliations:** ^1^ Division of Endocrinology, Metabolism and Bone and Mineral Disorders, Henry Ford Health, Detroit, MI, United States; ^2^ Department of Endocrinology, Postgraduate Institute of Medical Education and Research (PGIMER), Chandigarh, India; ^3^ Department of Pathology and Laboratory Medicine, Henry Ford Health, Detroit, MI, United States; ^4^ Bone and Mineral Research Laboratory, Henry Ford Health, Detroit, MI, United States

**Keywords:** large parathyroid adenomas, atypical parathyroid adenomas, giant parathyroid adenoma, primary hyperparathyroidism, osteitis fibrosa cystica, vitamin D nutrition, vitamin deficiency

## Abstract

Parathyroid adenomas weighing more than 3.5 g are reported variously as “atypical”, “large” or “giant” parathyroid adenomas. All such adenomas are rare variants accounting for no more than 1.5% of all parathyroid adenomas. Large parathyroid adenomas are often associated with more severe form of the disease, including osteitis fibrosa cystica (OFC) and share many biochemical, histological, and molecular features of both benign and malignant parathyroid neoplasms, and are considered a distinct clinical entity. However, the pathogenesis of oversized parathyroid adenomas and the often-associated skeletal phenotype remains unclear. We present 5 cases of primary hyperparathyroidism (PHPT) with OFC, an uncommon manifestation of contemporary PHPT, associated with larger parathyroid adenomas, seen in the Bone and Mineral Disorders Clinic of the Henry Ford Health in the last 30 years to illustrate the critical role of vitamin D nutrition in the pathogenesis of both the OFC and adenoma size. The estimated prevalence of OFC was very low 0.2%, 5 of the >3000 surgically confirmed cases of PHPT seen during this time. The mean ± SD values were: age: 36.8 ± 22.1 years (4 of the 5 <36years), serum calcium 11.6 ± 1.1 mg/dl, alkaline phosphatase 799 ± 487 IU/L, PTH 1440 ± 477 pg/ml, 25-hydroxyvitamin D 13.0 ± 8.9 ng/ml, 1,25-dihyroxyvitamin D 26.5 ± 13.7 pg/ml, urine calcium 562 ± 274 mg/day, and parathyroid adenoma weight 4.53 ± 2.2 g. Parathyroidectomy led to the resolution of both the biochemical indices and OFC in each patient without recurrence over >10 years of follow-up. Because OFC is a very rare in the West, but very common areas of endemic vitamin D deficiency, we also examined the relationship between vitamin D nutrition, as assessed by serum 25-hydroxyvitamin D level, and parathyroid adenoma weight as well as prevalence of OFC in two large secularly diverse cohorts of patients with PHPT (Detroit, USA and Chandigarh, India). Based on this relationship and the relative prevalence of OFC in these two large cohorts, we propose that vitamin D nutrition (and perhaps calcium nutrition) best explains both the adenoma size and prevalence of OFC.

## Introduction

Oversized adenomas are rare variants of sporadic parathyroid adenomas and have been variously reported as “atypical, “large” or “giant” parathyroid adenomas (1–3). All such adenomas are rare variants of sporadic parathyroid adenomas and the prevalence ranged from 0.5 – 4.4% among consecutive series of patients undergoing parathyroidectomy (4). In the 2022 WHO definition, atypical parathyroid adenomas are now reclassified as atypical parathyroid tumors in view of their potential for malignant behavior, and account for most of the oversized parathyroid adenomas. “Giant” parathyroid adenomas are even rarer with only 65 cases reported in the literature with variable clinical and biochemical manifestations (1). In contrast, large parathyroid adenomas are often associated with more severe form of the disease, including osteitis fibrosa cystica (OFC) and share many biochemical, histological, and molecular features of both benign and malignant parathyroid neoplasms, and are considered a distinct clinical entity (5). However, the pathogenesis of oversized parathyroid adenomas and the often-associated skeletal phenotype remains unclear.

Among these 3 variants, atypical parathyroid adenomas (now reclassified as atypical parathyroid tumors) are the most studied because of their shared histological and genetic features with benign and malignant parathyroid tumors (4). In an earlier era, osteitis fibrosa cystica (OFC), the specific bone lesion due to excess parathyroid hormone (PTH) secretion was an important diagnostic clue to the presence of primary hyperparathyroidism (PHPT) and was almost always associated with large parathyroid adenomas and vitamin D and calcium deficiency (6–9). OFC also occurs in both renal and non-renal secondary hyperparathyroidism (10, 11). In severe cases, cystic enlargement with bone deformities, referred to as brown tumors, that mimic the radiologic features of metastatic disease, bone cysts and giant-cell tumors can occur (12, 13), but these are not true bone neoplasms.

In the modern era, PHPT typically presents as an asymptomatic disorder of non-progressive mild hypercalcemia, mostly in the west (14–16). However, OFC continues to be a predominant manifestation in a sizable proportion of PHPT patients seen in parts of the world where vitamin D and calcium deficiency is endemic (17, 18). Before 1935 (19), and well into the 1960s (20, 21), almost all patients with PHPT had OFC, which declined to about 30% by the late 1960s and further down in the late 1980s (22). The dramatic simultaneous decline in the prevalence of both OFC and parathyroid adenoma size coincided with improvements in vitamin D and calcium nutrition of the population (22, 23), analogous to the disappearance of endemic goiter after iodination of common salt. Nevertheless, large parathyroid adenomas are still seen and thought to represent a distinct clinical entity sharing the histological features of both benign and malignant parathyroid tumors (1, 2, 24). However, the pathogenesis and disease phenotype of APA and other oversized variants of parathyroid tumors remains unclear. Several molecular mechanisms have been proposed, but collectively these abnormalities are seen in only a fraction of large parathyroid tumors (3).

We present 5 cases of PHPT in whom OFC was the principal presenting manifestation of the disease associated with larger parathyroid adenomas. Parathyroidectomy resulted in clinical, biochemical, and radiological improvement in each patient. Since OFC and large parathyroid tumors are more common in patients with PHPT living in parts of the world with endemic vitamin D deficiency, we also examined the relationship between vitamin D nutritional status as assessed by serum 25-hydroxyvitamin D level, the best available index of vitamin D nutrition, and parathyroid adenoma size in two large secularly diverse cohorts of PHPT patients (Detroit, USA and Chandigarh, India). Based on this relationship, we propose a unifying mechanistic concept that vitamin D nutrition (and perhaps calcium nutrition) best explains both the adenoma size and prevalence of OFC, and that the observed genetic mutations are likely to be secondary to increased cell proliferation imposed by demand for higher PTH secretion.

## Materials and methods

### The cases and the cohorts

The 5 patients (3 women and 2 men; 3 Caucasians, 1 middle Eastern and 1 African American) with OFC seen in the Bone and Mineral Clinic of Henry Ford Health System were part of >3000 PHPT patients seen over 30 years and represented approximately 0.002% of all patients with surgically confirmed sporadic PHPT without recurrence. Details of cases are described briefly in the next section and relevant laboratory data are summarized in **Table 1**.

**Table 1:** Relevant biochemical and parathyroid adenoma weights in the 5 cases with Osteitis Fibrosa Cystica (OFC) from Detroit.

Varaiable Measured	Case-1	Case-2	Case-3	Case-4	Case-5
Age (Years)	75	27	36	20	25
Serum Ca (mg/dl)	13.1	11.1	10.2	11.1	12.4
Serum Phosphate (mg/dl)	3.8	2.8	2.2	3.1	2.0
Serum Creatinine (mg/dl)	2.7	1.3	1.4	1.1	0.62
Serum 25-OHD (ng/ml)	23	2.0	7.0	12	21
Serum AP (IU/ml)	378	725	897	1580	416
Urine Calcium (mg/day)	ND	456	ND	357	873
Serum Parathyroid Hormone (pg/ml)	2188	1256	1352	1517	889
Parathyroid Adenoma Weight (g)	2.8	6.8	6.0	2.5	4.0

ND, Not done.

Reference Ranges: Serum Calcium 8.0-10.1mg/dl; Serum Phosphorus 2.5-4.5 mg/dl; Serum Creatinine 0.6-1.5 mg/dl; Serum 25-OHD >15ng/ml; Serum AP <140 IU/L; Urine Calcium 100-300 mg/day; and Serum PTH 10-75 pg/ml.

To further explore and examine the relationship between parathyroid adenoma size and vitamin D nutrition, we selected two secularly diverse cohorts of PHPT patients with different prevailing vitamin D nutritional status. The Detroit. USA cohort consisted of 429 PHPT patients with surgically confirmed adenoma size of ≥100 mg with subsequent cure of the disease without recurrence. They were part of larger ongoing study examining the relationship between vitamin D nutrition and adenoma size (7, 25). The Chandigarh, India cohort consisted of 426 patients who were part of an ongoing longitudinal study of PHPT from the Indian PHPT Registry (17, 18). Some aspects of both cohorts have been published, but in this study, we explored 3 aspects of the disease: 1) the proportion of parathyroid adenoma variants in the 2 cohorts and as a function of vitamin D nutritional status within each cohort; 2) the prevalence of OFC in the 2 cohorts and how these 5 patients from the Detroit cohort differed from the rest within that cohort; 3) the relationship between adenoma size and prevailing vitamin D nutritional status, as assessed by the serum 25-hydroxyvitamin D (the best available index of vitamin D nutrition) as well as the relationship between serum PTH levels and adenoma size.

### Laboratory methods

Serum levels of total calcium (Ca; reference range (RR): 8.2–10.0 mg/dL), phosphate (P; RR: 2.5–4.5 mg/dL), alkaline phosphatase (AP; RR: 0–120 IU/L), Creatinine (Cr; RR: 0.6–1.5 mg/dL), total protein, and albumin were measured in the hospital laboratory by standard methods using a Hitachi-747 autoanalyzer (Hitachi, Hialeah, FL). Serum Ca was adjusted for serum albumin as previously reported (26, 27). Serum intact PTH (reference range, 10–65 pg/mL) was measured by immunoradiometric assay (Nichols Institute Diagnostics, San Juan Capistrano, CA). Serum 25hydroxyvitamin D was measured by RIA (INCSTAR Corp., Stillwater, MN; RR: 15−60 ng/mL). The coefficient of variation for repeat measurements was 10% for 25-hydroxyvitamin D and 5% for PTH. Although methods to measure serum PTH and 25-hydroxyvitamin D have changed over the years, the reference ranges remained relatively similar. Also, the laboratory methods used in the Indian cohort are similar to those used in the Detroit cohort and the details have been published (17, 18).

Adenoma size was defined as large or giant based on previously suggested cutoff value of 3.5g, which is above the 95^th^ percentile of all sporadic parathyroid adenomas (5), and close to the median adenoma weight of 3.8g for the atypical parathyroid tumors (4). Giant adenomas are defined as >10g for both the cohorts. The relationship between parathyroid adenoma weight and serum 25-hydroxyvitamin D level was examined in each cohort as previously demonstrated in 2 independent cohorts of PHPT patients from Detroit, but without OFC (7, 25). Finally, we examined the prevalence of OFC in both cohorts and in the sub-set of large and small adenomas as defined (5).

### Case descriptions

#### Case 1

A 75-year-old Caucasian dentist presented with a 4-year history of hypercalcemia, bone pain and muscle weakness. Hypercalcemia due to PHPT was diagnosed initially at the time of cholecystectomy 4 years ago. He had one failed parathyroid exploration with persistent hypercalcemia ranging between 11-13 mg/dl with serum PTH levels >2000 pg/ml. Four years prior to presentation, he developed progressive muscle weakness and became wheelchair bound. He also had polyuria, polydipsia, nausea, and constipation. Two months prior to presentation, he developed severe right arm pain while attempting a tooth extraction on his patient. X-rays showed a lytic lesion in the forearm and a subsequent biopsy was consistent with OFC. X-rays of his hands showed cortical thinning, sub-periosteal resorption in the phalanges and lytic lesions in the right thumb and radius. Serum biochemical measurements were as shown in **Table 1**. A technetium sestamibi parathyroid scan localized a single left inferior parathyroid adenoma. At surgery, a parathyroid adenoma weighing 2.88g was removed. Post operatively, serum PTH levels decreased from 2188 pg/ml to 4 pg/ml and serum Ca decreased from 13.1 to 8.0 mg/dl and returned to within the reference range in 2 months. Three months after surgery, his symptoms improved and within the next year, his hormonal and biochemical indices were within the reference range. Repeat X-rays showed resolution of the lytic lesions in the thumb and radius. There was no recurrence of hypercalcemia over >10 year follow-up.

#### Case 2

A 27-year-old African American woman presented with diffuse bone pain of 6-9 months duration. Incidental hypercalcemia was detected, but she had no symptoms related to this. She had chronic low back pain which prompted imaging that revealed bilateral lytic lesions in sacral alae and iliac wings. Because of the lytic lesions and hypercalcemia, she was evaluated for metastatic cancer with further imaging and even a biopsy of a suspicious cervical lymph node, which was benign. A bone scan showed diffuse uptake of radiotracer, greatest in the calvarium, sternum and appendicular skeleton. Relevant biochemical measurements are in **Table 1**. At neck exploration, a 6.82 g left inferior parathyroid adenoma was removed. Follow-up x-rays a few months later, showed healing of OFC and normalization of biochemical measurements without recurrence over 20 years.

#### Case 3

A 36-year-old Caucasian woman with a history of cystic fibrosis was seen in the bone and mineral clinic for evaluation of diffuse bone pain and muscle weakness. She had well controlled cystic fibrosis and was taking pancreatic enzyme supplements, thyroid hormone replacement and insulin for cystic fibrosis associated diabetes. A bone scan showed increased uptake in a few ribs and in the distal right fibula. Laboratory evaluation showed a serum calcium of 10.2 mg/dl and a PTH level of 1352 pg/ml. Correlative x-rays revealed evidence of OFC. A trans-iliac bone biopsy showed an unusual combination of osteomalacia and bone marrow fibrosis. She was sent for surgery after pretreatment with vitamin D, and a 6-g parathyroid adenoma was removed. Postoperative course was complicated by hungry bone syndrome, requiring calcitriol and calcium therapy for one year following which the biochemical measurements normalized associated with clinical improvement. There was no recurrence of hypercalcemia over >15 year follow-up.

#### Case 4

A 20-year-old Caucasian male was seen in the bone and mineral clinic after his endocrinologist sent him for evaluation of PHPT. He developed bone pain in his right lower extremity, a year prior to evaluation. He subsequently developed a mass in that region which showed a cystic lesion on imaging. Initially, his labs showed a serum calcium of 11.1 mg/dl, PTH of 1517 pg/ml, AP of 2395 IU/L, undetectable 25, hydroxy vitamin D and a 24-hour urine calcium of 357 mg/vol. He was treated with Vitamin D, calcitriol and cinacalcet, prior to parathyroidectomy. He also had further skeletal imaging, which showed multiple cystic lesions in bilateral femur, tibia, right shoulder, and several long bones, as well as subperiosteal erosion in the symphysis pubis. At surgery, a 2.5 g right superior parathyroid adenoma was removed. His biochemical indices normalized in a few months and bone lesions resolved in one year. There was no recurrence of hypercalcemia over >15 year follow-up.

#### Case 5

26-year-old Middle Eastern female was seen in the bone and mineral clinic for evaluation of hypercalcemia. She is an avid runner and developed ankle and knee pain, which prompted imaging revealing a lytic lesion in the distal medial femur. Subsequently a manetic resonance image (MRI) showed T1 isointense, T2 hyperintense lesions within the ventral patella, left acetabulum, and medial femoral condyle. Biochemical testing showed an elevated serum calcium of 12.4 mg/dl with a concomitant PTH of 889 pg/ml. She had recently started vitamin D supplementation because of knee pain and her serum 25-hydroxyvitamin D level was 21 ng/ml. Neck imaging showed a large mass along the right thyroid lobe and at surgery, she required a right thyroid lobectomy as the gland was partially intrathyroidal. The parathyroid mass was 2.1 cm in size but could not be weighed separately, being intra-thyroidal, and so an estimated weight of 4.0g was assigned based on tumor dimension. Postoperatively, calcium levels normalized, and subsequent imaging at 6 months showed healing of lytic lesions. There was no recurrence of hypercalcemia over >5 year follow-up.

### Statistical methods

Descriptive statistics are shown as mean ± SD or as proportions. Group comparisons were examined by unpaired t-test or ANOVA as appropriate, and the difference in proportions by Chi squared test with Yate’s corrections for small sample size as necessary. Frequency distribution of adenoma weight was inspected by histograms in both cohorts, but the data for the relevant variables was non-normally distributed. The relationship between adenoma size and vitamin D nutrition was examined after log-transforming adenoma weights, and serum levels of 25-hydroxyvitamin D and PTH as appropriate because of non-normal distribution. The relationship was assumed to be linear as previously reported (7, 25) The differences in the slopes of regressions in the 2 cohorts was tested with least square means. A p value of <0.05 was considered significant for all the differences. We used Sigma Plot version 14.5 for all statistical analyses. The data were collected in an Excel file and then downloaded to Sigma Plot.

## Results

**Figure 1:**
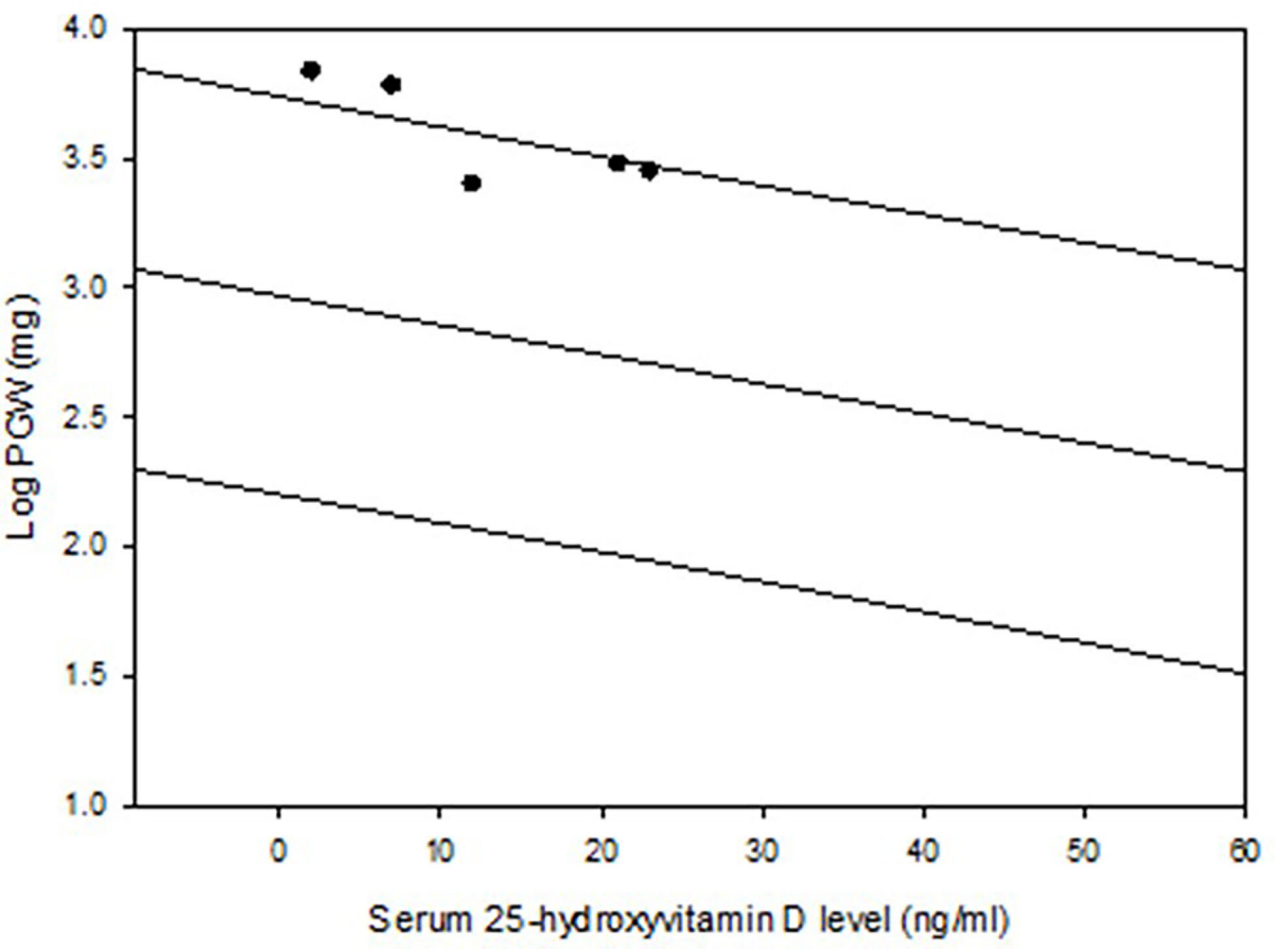
Regression and 95% prediction interval of the relationship between serum 25-hydroxyvitamin D level and parathyroid adenoma weight in the Detroit cohort (n=429). The 5 patients with osteitis fibrosa cystica are depicted (closed dots). Note the location of these 5 adenomas near or above the predicted interval.

The detailed characteristics of the two cohorts are summarized in **Table 2**. The characteristics of the Indian cohort have been reported in other publications (17, 18), but the data is used here for comparative analyses. Frequency distribution of adenomas size was unimodal in both cohorts but was skewed to the left with more smaller adenomas (data not shown). The Chandigarh, India cohort was almost 2 decades younger, had more severe disease indices, higher prevalence of OFC, larger and giant adenomas, and cancers than the Detroit, USA cohort (**Table 2**). As can be expected, mean serum levels of Ca, AP, and PTH were higher and serum P and 25-hydroxyvitamin D levels were lower in patients with larger adenomas with significant difference *within* and *between* cohorts when compared to the smaller adenomas (**Table 3**). However, patients with larger adenomas in the Detroit cohort were younger compared to those with smaller adenomas (56.5 ± 16.5y Vs. 62.0 ± 13.0y; p<0.001, **Table 3**), but such age difference was not observed in the Chandigarh, India cohort. Three of the 5 patients with OFC in the Detroit cohort had large adenomas as defined, all had vitamin D deficiency or insufficiency, and the adenoma size was near or above the 95% prediction interval for the entire Detroit cohort (**Figure 1**). The prevalence of large adenomas was 35% in the Indian cohort, but only 7% in the Detroit cohort with significant difference between the cohorts (p<0.001; **Table 2**). Although the sample sizes in both cohorts was small, the prevalence of parathyroid cancers and giant adenomas (data not shown) was higher in the Indian compared to the Detroit cohort (0.2% Vs. 1.7%; p=0.038) despite younger age of the Indian cohort (43.3 ± 13.9 Vs. 61.6 ± 13.2y; p<0.001, **Table 2**).

**Table 2:** Comparison of biochemical and parathyroid adenoma characteristics and the prevalence of osteitis fibrosa cystica in the two secularly diverse cohorts (Detroit, USA and Chandigarh, India).

Variable Measured	Detroit Cohort	Indian Cohort	p values
Sample Size (n)	429	426	
Age (Years)	61.6 ± 13.2	43.3 ± 13.9	< 0.001
Serum Ca (mg/dl)	11.0 ± 0.82	11.9 ± 1.98	< 0.001
Serum 25-OHD (ng/ml)	20.4 ± 9.46	15.8 ± 8.18	< 0.001
Serum AP (IU/ml)	112 ± 82	352 ± 737	< 0.001
Serum Parathyroid Hormone (pg/ml)	162 ± 215	684 ± 748	< 0.001
Parathyroid Adenoma Weight (g)	1.26 ± 2.63	4.53 ± 6.65	< 0.001
Large Adenomas (%)	28 (6.5)	150 (35.2)	< 0.001
Small Adenomas (%)	401 (93.5)	276 (64.8)	
Patients with Parathyroid Cancers (%)	1 (0.23)	7 (1.64)	< 0.001
Patients without Parathyroid Cancers (%)	428 (99.8)	419 (98.4)	
Patients with OFC (%)	7 (1.63)	97 (22.8)	< 0.001
Patients without OFC (%)	422 (98.4)	329 (77.2)	
OFC Patients with Large Adenomas (%)	4 (0.93)	54 (12.7)	< 0.001
Non-OFC Patients with Large Adenomas (%)	425 (99.1)	372 (87.3)	
OFC Patients with Small Adenomas (%)	3 (0.70)	43 (10.1)	< 0.001
Non-OFC Patients with Small Adenomas (%)	426 (99.3)	383 (89.9)	

Data shown as mean ± SD or as numbers and proportions.

**Table 3:** Comparison of age, biochemical, and adenoma characteristics by adenoma type in the Detroit, USA and Chandigarh, India cohorts (Data shown as mean ± SD).

Variable Measured	Detroit Large Adenomas	Detroit Small Adenomas	p values	Chandigarh Large Adenomas	Chandigarh Small Adenomas	p values
Sample Size	28	401		282	144	
Age (years)	56.5 ± 16.5	62.0 ± 13.0	< 0.001	42.3 ± 14.5	43.8 ± 13.5	NS
Serum Calcium (mg/dl)	11.9 ± 1.26	11.1 ± 0.73	< 0.001	12.4 ± 2.33	11.6 ± 1.59	< 0.001
Serum Phosphate (mg/dl)	2.43 ± 0.55	2.86 ± 0.52	< 0.001	2.8 ± 1.0	2.66 ± 0.99	NS
Serum Creatinine (mg/dl)	0.94 ± 0.29	0.95 ± 0.58	NS	N/A	N/A	N/A
Serum Alkaline Phosphatase (IU/L)	192 ± 181	105 ± 54	< 0.001	462 ± 643	295 ± 776	0.019
Serom25-hydroxyvitamin D (ng/ml)	13.6 ± 8.2	20.7 ± 9.1	< 0.001	14.7 ± 7.5	16.6 ± 8.3	0.042
Serum Parathyroid Hormone (pg/ml)	471 ± 471	135 ± 140	< 0.001	1045 ± 860	499 ± 606	< 0.001
Parathyroid Adenoma Weight (g)	7.58 ± 7.62	0.82 ± 0.71	< 0.001	10.2 ± 9.0	1.65 ±0.97	< 0.001

Data are presented as mean ± SD. NS, Not significant; N/A, Not available.

When the 2 cohorts were sub-divided by the level of serum 25-hydroxyvitamin D of ≤20 ng/ml or >20 ng/ml, the prevalence of large adenomas was significantly lower in the Detroit cohort in patients with serum 25-hydroxyvitamin D ≤20 ng/ml (9% Vs. 53%; p<0.001, **Table 4**). In contrast, the prevalence of large adenomas was still lower in the Detroit cohort with serum 25-hydroxyvitamin D level of >20ng/ml compared to the Indian cohort (3% Vs. 44%; p<0.001, **Table 4**). However, the prevalence of large and small adenomas was about the same in the Indian cohort regardless of serum 25-hydroxyvitamin D level (large: 53% and 44%; small: 48% Vs. 56%; **Table 4**). This implies that there is an additional effect of low calcium intake and other unidentified factors on the adenoma size in the Indian cohort (28).

**Table 4:** Prevalence of large and small parathyroid adenomas in the two secularly diverse cohorts (Detroit, USA and Chandigarh, India) stratified by serum 25-hydroxyvitamin D levels.

Variable	Detroit Cohort		p values	Indian Cohort		p values
	25-OHD<20	25-OHD >20		25-OHD<20	25-OHD >20	
Sample Size (n)	236	193		204	222	
Age (Years)	60.6 ± 13.8	62.9 ± 12.4	< 0.01	40.6 ± 13.6	45.7 ± 13.7	< 0.01
Serum 25-OHD (ng/ml)	13.7 ± 4.7	28.6 ± 7.2	< 0.01	10.8 ± 5.0	25.2 ± 3.0	< 0.01
Serum Parathyroid Hormone (pg/ml)	187 ± 225	132 ± 199	< 0.01	700 ± 725	669 ± 769	NS
Parathyroid Adenoma Weight (g)	1.56 ± 3.32	0.89 ± 1.31	< 0.01	4.4 ± 5.0	4.7 ± 7.9	NS
Patients with Large Adenomas (>3.5 g)	22 (9.3%)	6 (3.1%)	0.013	107 (52.5%)	98 (44.1%)	0.086
Patients with Small Adenomas (<3.5 g)	214 (90.3%)	187 (96.4%)		97 (47.5%)	124 (55.9%)	

When stratified by serum 25-hydroxyvitamin D levels, all the differences within and between cohorts was significant (p values for the between cohort differences not shown to avoid cluttering with additional data columns). The differences for the adenoma weights remained significant even after excluding giant adenomas (adenoma weight >10g).

**Figure 2:**
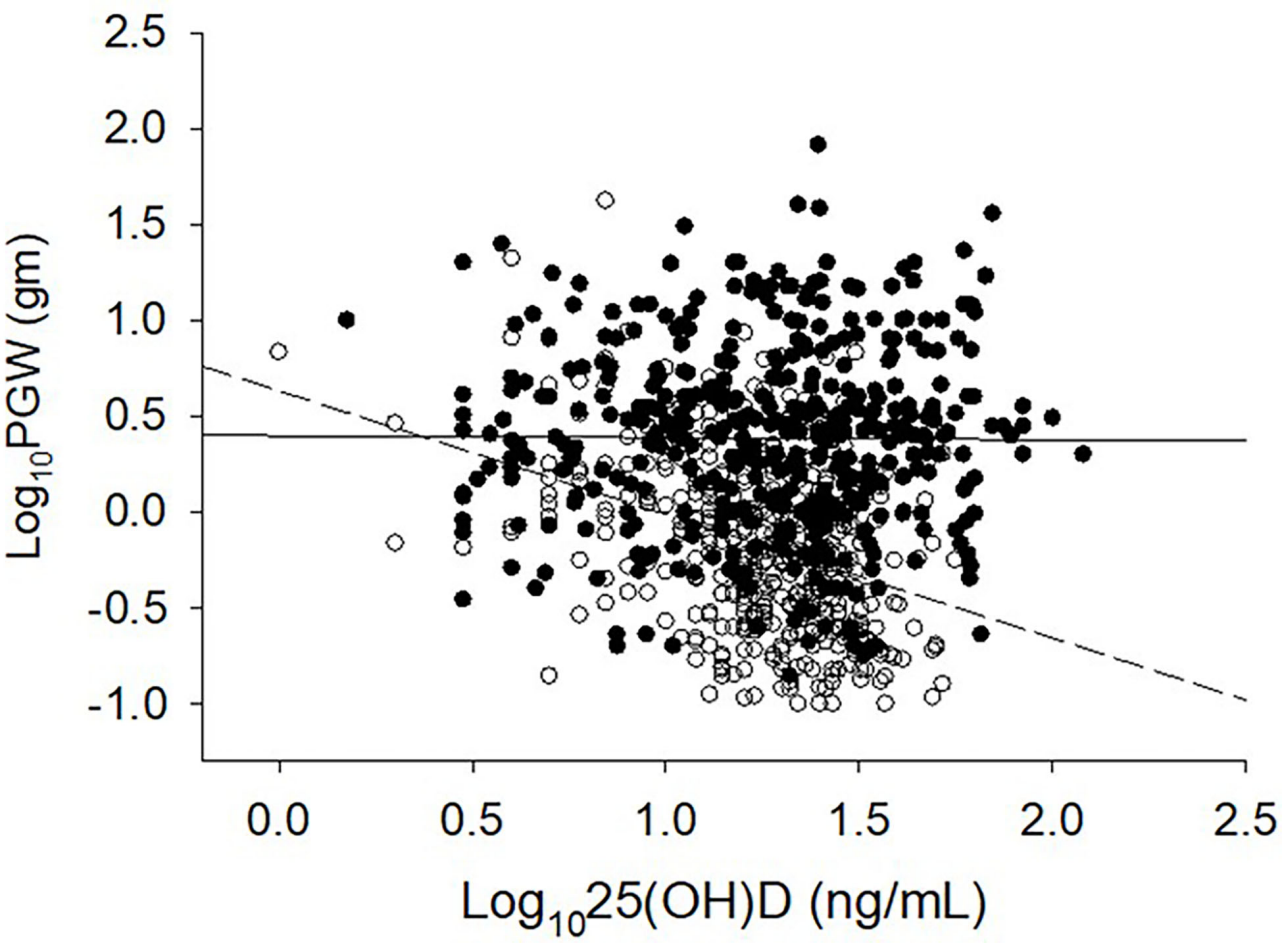
Relationship between serum 25-hydroxyvitamin D level and parathyroid adenoma weight in 2 large secularly diverse cohorts. Detroit Cohort: Open Circles and Dashed line (n=429). Indian Cohort: Closed Circles and Solid line (n=426). Data was log transformed because of the non-normal distribution of both variables. Note the lack of inverse relationship in and an upward shift of the Indian PHPT data.

**Figure 3:**
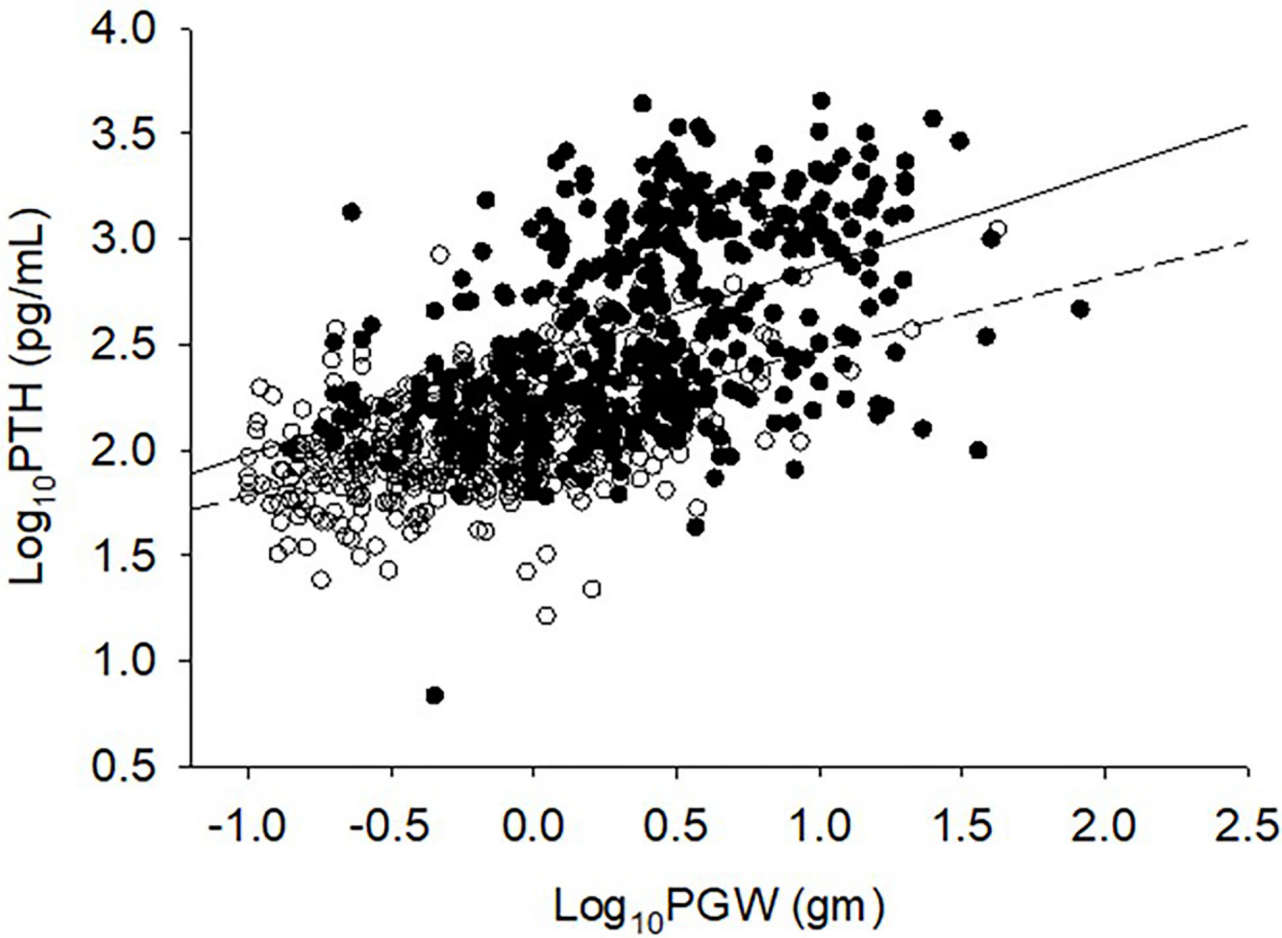
Relationship between serum PTH level and adenoma size. Detroit Cohort: Open circles and bottom solid line (n=426). Indian Cohort: Closed circles and top solid line (n=426). Data was log transformed because of non-normal distribution of both variables. Note the upward and rightward shift of Indian PHPT data. Slopes of regression are not different from each other, but the intercept is higher for the Indian cohort.

The relationship between adenoma size and serum 25-hydroxyvitamin D levels in the 2 cohorts are shown in **Figure 2**. There was an inverse relationship between the adenoma size and serum 25-hydroxyvitamin D level in the Detroit cohort similar to our previous reports (7, 25), but not in the Indian cohort. Also, for any given level of serum 25-hydroxyvitamin D level, Indian PHPT patients had larger adenomas compared to the Detroit PHPT patients, which may explain the lack of expected inverse relationship between these two variables in the Indian cohort or obscured by the additional effect of low calcium intake in the Indian population (28). There appeared be an overall upward shift of adenomas in the Indian cohort compared to the Detroit cohort (**Figure 1**). By contrast, the relationship between serum PTH level and adenoma weight was similar in both cohorts with no difference in the slopes of regression (**Figure 3**), but the intercept was higher in the Indian cohort implying that for any given adenoma weight PTH secretion was higher in the Indian PHPT patients (**Figure 3**). Also, the relative slopes of regression lines suggests that larger tumors secrete relatively less PTH/g adenoma weight than smaller adenomas. There appeared be an overall upward and rightward shift of adenomas in the Indian cohort (**Figure 3**).

Finally, we examined the prevalence of osteitis fibrosa cystica (OFC), the specific bone lesion of PHPT, in both cohorts. The 5 patients described in detail were the only patients with OFC seen in Detroit cohort: a prevalence of 1.2%, but the total prevalence of OFC was 0.2% (5 of >3000) PHPT patients seen over a 30-year period. Of these 5 patients, 3 had large and 2 had small adenomas as defined. This is in stark contrast with the Indian cohort in which the prevalence of OFC was 22.8% (or 97 of 426), with 54 large and 43 small adenomas associated with OFC. As expected, the difference in the prevalence of OFC was highly significant (p<0.001).

## Discussion

In earlier times there was a widely held belief that different degrees of disease severity was caused by the same hormonal abnormality with very little overlap between these predominant types of presentation (19, 21, 29). Because each type remained true to the type of initial presentation and did not change or progress from one form to the other, Lloyd concluded that there were 3 principal types of PHPT: Group-1 with predominantly bone disease, Group-2 with predominantly stone disease and a small group (Group-3) without bone or stone disease based on detailed analysis of cases and the role of parathyroid tumor in the phenotypic expression of the disease (20). In contemporary times, the non-specific skeletal effects of PTH excess such as cortical thinning, low bone mass and osteoporosis are more common than the specific bone lesions such as subperiosteal bone resorption, brown tumors, OFC, and pathological fractures. The disease usually presents as mild asymptomatic hypercalcemia discovered during routine biochemical testing (30–32). A simultaneous steep decline both in parathyroid adenoma size and prevalence of OFC, but a steady rise of age at presentation is commonly attributed to the introduction of routine biochemical screening with consequent earlier detection and diagnosis. Although this explains the relative decrease in bone phenotype, not the absolute decrease (13, 22, 23).

The concept of large and small parathyroid adenomas is not really new and was first examined in detail as early as 1968, which was the basis for Lloyd to classify PHPT phenotypes into 3 discrete groups (20). Patients in Group-1 (or Lloyd Type-1 PHPT) had bone disease without stone disease, higher serum Ca, larger tumor weights and shorter duration of symptoms, whereas patients in group-2 (or Lloyd Type-2 PHPT) had predominantly stone disease without the bone disease, lower serum Ca, smaller tumor weights, and longer duration of symptoms (20). The Lloyd concept was further expanded in a modified model by Rao, Bhadada, and Parfitt to explain the dichotomous tumor behavior driven by either dysfunction in calcium set-point control or in cell-cycle control (13). According to this modified model, somatic mutations found in both sporadic benign and malignant parathyroid tumors are more likely in Lloyd Type-1 than in Type-2 PHPT (13). Unfortunately, serum PTH or 25-hydroxyvitamin D measurements were not available at the time of Lloyd’s study (20). Furthermore, the relationship between adenoma weight and biochemical phenotype *in vivo*, and secretory capacity of large and small parathyroid tumor cells invitro, was first reported in 1992 (33). Although there was no relationship either between serum Ca and PTH or between serum Ca and adenoma weight, there was a highly significant correlation between serum PTH level and adenoma weight (33). Interestingly, larger parathyroid tumors secreted less PTH in relation to adenoma weight compared to the smaller tumors, which was confirmed in a small sub-set of parathyroid tumor cells large and small tumors, but no data was reported on vitamin D nutritional status of the study population (33).

We first reported the significant inverse relationship between serum 25-hydroxyvitamin D level and adenoma weight in a small sub-set of patients with PHPT seen in Detroit (25) and recently confirmed in a much larger number of patients (7). We also reported the potential role of calcium and vitamin D nutrition on serum PTH levels and adenoma weights from different parts of the world (22). In the current study, we further demonstrate the effect of vitamin D nutrition, as assessed by serum 25-hydroxyvitamin D level, the best available index of vitamin D nutrition, and adenoma weight in 2 large secularly diverse cohorts of large number of patients with PHPT. In addition, we studied the distribution of small and large tumors in these 2 cohorts as defined (5). In neither cohort was there any bimodal distribution of adenoma weights, which suggests that the proposed cut-off to define small, large and giant adenomas parathyroid adenomas appears rather arbitrary (5). Although molecular studies of atypical parathyroid tumors demonstrated several somatic mutations in cell-cycle control genes, but such molecular signatures account for only a small fraction of both sporadic, small, large, or giant parathyroid adenomas, including parathyroid tumors (2, 3).

Another interesting observation is the temporal change in bone phenotype and dramatic decline in its prevalence in patients with PHPT seen in the west over the past century (8, 9, 22). This steep decline in bone phenotype occurred despite a 3-10-fold increase in the incidence of PHPT in the past 50 years in the Western world (30–32, 34). However, such change in the prevalence of bone phenotype is not seen in parts of the world where vitamin D and calcium nutrition is inadequate or insufficient (8, 9, 22, 23). Earlier diagnosis and treatment are often mentioned as reasons for the rarity of OFC in patients with modern-day PHPT seen in the West, but long-term follow up without parathyroidectomy is associated with neither worsening biochemical indices nor the development of OFC (14–16). In fact, the mean age at diagnosis of PHPT increased significantly in the West, but not in parts of the world with endemic vitamin D deficiency where OFC is still a predominant manifestation of PHPT (8, 9, 22, 23).

One plausible explanation for the wide variation in disease phenotype, parathyroid adenoma weight, serum PTH levels, and prevalence of bone disease from different parts of the world, as well as for the apparent decline in OFC over time in the west could be related to vitamin D status of the population. In studies from USA, UK, Denmark, France, and other countries, patients with PHPT with radiographic OFC had significantly lower serum levels of 25-hydroxyvitamin D and/or 1,25-dihydroxyvitamin D than patients with normal skeletal X-rays (7, 22, 25, 35–39). The apparent lack of inverse relationship between serum 25-hydroxyvitamin D level and adenoma weight in the Indian cohort (**Figure 3**) is a bit intriguing but could be related to nutritional calcium deficiency and other unidentified factors affecting parathyroid adenoma growth and behavior. The pattern of relationship between adenoma weight and serum PTH level (with similar slopes but with different intercepts; **Figure 3**) supports our hypothesis. Further detailed studies are needed to confirm our speculation.

Four of the 5 patients from Detroit in this study had low serum 25-hydroxyvitamin D levels that could have contributed both to the development of large parathyroid adenomas and OFC. A normal serum 25-hyroxyvitamin D level in patient 5 could have been related to recent vitamin D supplementation. The mean parathyroid gland weight in these patients with vitamin D deficiency was similar to that seen in patients with PHPT and OFC reported from India, as well as to patients with OFC seen in the US and UK at an earlier time. Lack of four gland involvement in all the patients with PHPT reported here and in Indian cohort, suggests that vitamin D deficiency was not involved in the genesis of parathyroid adenoma but rather to the increased growth rate of the parathyroid adenoma, which in turn contributes to the development of OFC through increased demand for PTH secretion.

**Figure 4:**
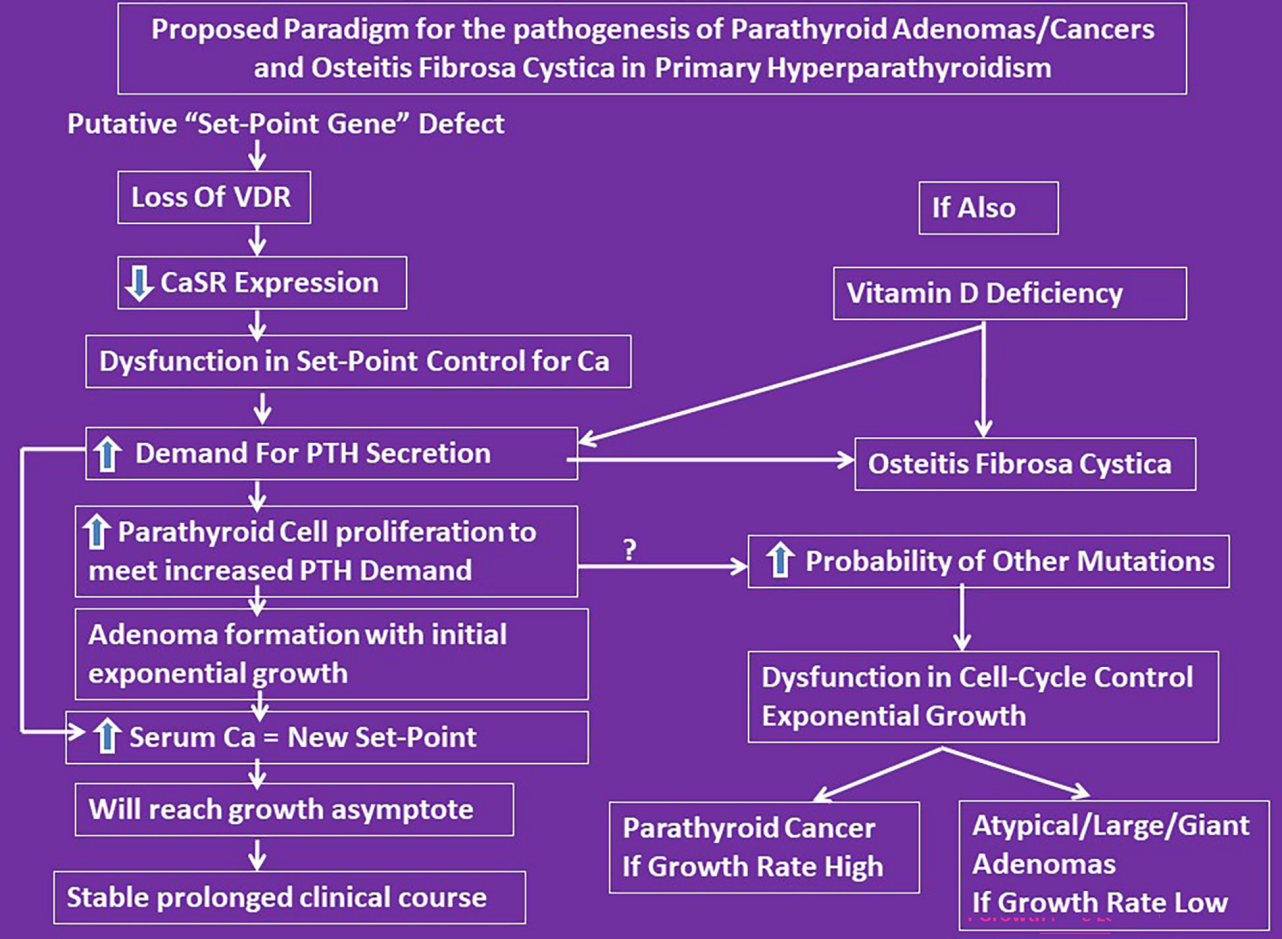
Proposed conceptual mechanistic explanation for the pathogenesis of both large adenomas and osteitis fibrosa cystica. It reconciles the Lloyd hypothesis of Type-1 and 2 PHPT and the Parfitt-Rao proposal of set-point and cell-cycle dysfunctions leading to different types of adenoma formation. However, we do yet not know what genes control the set-point. According to this concept, the observed genetic and epigenetic abnormalities are consequences of set-point dysfunction as the initial event.

We propose a unifying hypothesis implicating vitamin D nutrition to explain both the accentuated parathyroid tumor growth leading to large adenomas, and the steep decline in adenoma weight and prevalence of OFC within a region over time and contemporaneous differences in these characteristics between regions (**Figure 4**). This is analogous to the decline in endemic goiter after the introduction of iodized salt. Similarly, the occurrence of OFC will be restricted to those patients who are vitamin D deficient or to parts of the world where vitamin D deficiency is endemic. In this context, it is instructive to recall Fuller Albright’s brilliant prediction about calcium nutrition and bone disease in PHPT 75 years ago (6). *“If the patient is in negative calcium balance, bone disease develops; if the patient happens to ingest sufficient calcium to compensate for the loss in the urine and feces, the calcium balance is not negative and bone disease does not develop. For all practical purposes it usually comes down to whether or not the patient drinks milk. If he does, the calcium intake will be sufficient to keep a positive calcium balance even if he has marked hyperparathyroidism*”. No compelling data have been presented since to alter his prescient reasoning (6). Although he did not mention “vitamin D”, he most likely had this in mind as well (8, 9).

In summary, OFC is distinctly uncommon in patients with contemporary PHPT, but continues to occur in patients with coexistent vitamin D (and calcium) deficiency regardless of the geographic location. Changes in parathyroid adenoma weight and its behavior and the bone phenotype are largely dependent on these two key nutrients (28). The often-mentioned reasons for the change in clinical phenotype such as routine biochemical screening and early diagnosis are not supported by the data presented here and elsewhere in literature. Clinicians should not be deterred to promote optimal calcium and vitamin D nutrition, just as in the general population, for fear of worsening hypercalcemia. Recent meta-analysis has demonstrated the safety and efficacy of vitamin D repletion in patients with PHPT and vitamin D deficiency (40). Based on our detailed analysis of the 2 secularly diverse cohorts, other available evidence, and the experience of the senior author (SDR) at our institution over the past 50 years, it is suggested that *“not all patients with PHPT and vitamin D deficiency develop OFC, but OFC can occur only in those patients with PHPT who have prolonged Vitamin D deficiency”*. The precise role of somatic mutations observed both by us and others is primary or secondary to cell-cycle dysregulation imposed by an increased demand for PTH secretion due to vitamin D and calcium deficiency needs further clarification (2, 3, 41–48).

## Data availability statement

The raw data supporting the conclusions of this article will be made available by the authors, without undue reservation.

## Ethics statement

The studies involving human participants were reviewed and approved by Henry Ford Health and PGIMER. Written informed consent for participation was not required for this study in accordance with the national legislation and the institutional requirements.

## Author contributions

Concept, data acquisition, analysis, draft writing, editing, and final submission: SDR, AB, SKB, SQ, and SA. Data acquisition, analysis, editing, and final submission: All authors. All authors contributed to the article and approved the submitted version.
